# Solid-State Fermentation of Chestnut Shells and Effect of Explanatory Variables in Predictive Saccharification Models

**DOI:** 10.3390/ijerph19052572

**Published:** 2022-02-23

**Authors:** Paula A. Pinto, Rui M. F. Bezerra, Irene Fraga, Carla Amaral, Ana Sampaio, Albino A. Dias

**Affiliations:** 1 CITAB—Centre for the Research and Technology of Agro-Environmental and Biological Sciences, UTAD—Universidade de Trás-os-Montes e Alto Douro, 5000-801 Vila Real, Portugal; paulapinto@utad.pt (P.A.P.); bezerra@utad.pt (R.M.F.B.); ifraga@utad.pt (I.F.); camaral@utad.pt (C.A.); asampaio@utad.pt (A.S.); 2 Department of Biology and Environment, UTAD—Universidade de Trás-os-Montes e Alto Douro, 5000-801 Vila Real, Portugal

**Keywords:** chestnut shells, enzymatic hydrolysis, fungal pretreatment, waste valorization

## Abstract

In this study, chestnut shells (CNS), a recalcitrant and low-value agro-industrial waste obtained during the peeling of *Castanea sativa* fruits, were subjected to solid-state fermentation by six white-rot fungal strains (*Irpex lacteus*, *Ganoderma resinaceum*, *Phlebia rufa*, *Bjerkandera adusta* and two *Trametes* isolates). After being fermented, CNS was subjected to hydrolysis by a commercial enzymatic mix to evaluate the effect of fermentation in saccharification yield. After 48 h hydrolysis with 10 CMCase U mL^−1^ enzymatic mix, CNS fermented with both *Trametes* strains was recorded with higher saccharification yield (around 253 mg g^−1^ fermented CNS), representing 25% *w*/*w* increase in reducing sugars as compared to non-fermented controls. To clarify the relationships and general mechanisms of fungal fermentation and its impacts on substrate saccharification, the effects of some independent or explanatory variables in the production of reducing sugars were estimated by general predictive saccharification models. The variables considered were lignocellulolytic activities in fungal fermentation, CNS hydrolysis time, and concentration of enzymatic hydrolysis mix. Multiple linear regression analysis revealed a very high significant effect (*p* < 0.0001) of fungal laccase and xylanase activities in the saccharification models, thus proving the key potential of these enzymes in CNS solid-state fermentation.

## 1. Introduction

Lignocellulosic biomass from agricultural and forestry activities has been the object of increasing interest, due to decarbonization policies [1], as a renewable resource for the production of biofuels and value-added bioactive molecules [2,3,4,5,6]. During agro-industrial peeling of chestnuts (*Castanea sativa* Mill.), chestnut shells (CNS) were discarded as waste accounting approximately for 15% of the total weight of whole chestnuts [4,7]. Taking into account the requirements of the circular economy and the concept of biorefinery [8], CNS can contribute to the available range of renewable resources [6,8]. Morana et al. [8] reported that it is constituted of Klason lignin (41.7%) and carbohydrates (41.6%) including around 28.4% cellulose, 7.9% xylan, 0.3% cellobiose, 2.8% galactose, and 2.2% arabinose on a dry weight basis.

In order to improve the access to structural polysaccharides of lignocellulosic biomass and their hydrolysis, the deconstruction of a complex cell wall matrix is an imperative operation. Furthermore, this step, which is one of the most expensive unit operations in the bioconversion process [9], should be selective, with minimum loss of carbohydrates and favoring lignin removal. This recalcitrant heteropolymer of plant cell wall, hinders the enzymatic action, acting as a limiting factor of polysaccharides hydrolysis into fermentable sugars. Therefore, for an effective deconstruction of the recalcitrant cell wall matrix, lignocellulosic biomass must be submitted to pretreatment, especially when enzymatic hydrolysis is chosen for the saccharification process. Several physical, chemical, and biological pretreatments have been developed to improve enzyme accessibility to structural carbohydrates [10,11,12,13]. An efficient process for biomass pretreatment should preserve both carbohydrate fractions, pentoses (from hemicellulose) and hexoses (mainly from cellulose), without the formation of toxic products [13].

Biological pretreatments have been reported as promising eco-friendly tools due to several factors, such as low environmental impact, moderate reaction conditions, reduced side reactions and lower energy requirements [14]. More specifically, biological pretreatments aim to break down and remove the lignin “seal” and disrupt the crystalline structure of cellulose, increasing the susceptibility of the pretreated substrate to subsequent enzymatic or microbial attack, while minimizing the loss of carbohydrates [15]. Among biological pretreatments, solid-state fermentation carried out by some white-rot fungi (WRF), namely, *Phanerochaete chrysosporium*, *Pleurotus ostreatus*, *Trametes versicolor*, *Bjerkandera adusta*, *Ganoderma resinaceum*, *Irpex lacteus*, and *Phlebia rufa* have been studied in the pretreatment of agro-industrial residues such as wheat straw, cotton stalks, corn stover and grape stalks [5,12,14,15,16,17,18]. WRFs are able to depolymerize and mineralize lignin efficiently due to an extracellular and unspecific enzymatic system [12], and thus allow increasing saccharification yields of fermented biomass. In addition to ligninolytic enzymes, WRFs also produce extracellular carbohydrate-acting hydrolases such as cellulases, hemicellulases and xylanases which are also involved in the in vivo decaying process of lignocellulosic biomass. This pool of oxidative and hydrolytic enzymes constitutes a lignocellulolytic complex, whose mechanism of action and biochemical properties are well reviewed elsewhere [19]. Briefly, the ligninolytic system is composed mainly of laccase, lignin peroxidase (LiP), versatile peroxidase (*VP*; also called manganese independent peroxidase) and manganese-dependent peroxidase (*MnP*). While LiPs are capable of mineralizing lignin, recalcitrant aromatic pollutants and dyes, *MnP*s have a similar catalytic mechanism, but differ in utilizing Mn^2+^ as the primary electron donor. The *VP* has similar catalytic activities to LiP and *MnP*, i.e., it combines both peroxidase actions, and allows its application in Mn-mediated or Mn-independent reactions, with low or high redox potential aromatic substrates.

Although the potential of WRF enzymatic systems, aiming lignocellulosic biomass pretreatment and saccharification increase has already been studied, overall process mechanisms and the relationship between variables needs additional insights and/or further clarification. In this study, pretreatment of CNS by several fungal strains under solid-state fermentation was carried out and the production of extracellular lignocellulolytic enzymes complexes was evaluated. Subsequent enzymatic saccharification of pretreated CNS was quantified and the contribution of main factors namely, extracellular WRF enzyme activities, hydrolysis time and enzyme concentration, were estimated as components of saccharification yield. The contribution/influence of these factors was estimated through multiple linear regression models.

## 2. Materials and Methods

### 2.1. Pretreatment of Chestnut Shells by Fungal Solid-State Fermentation

Fruits (*Castanea sativa* Mill.) picked at the University of Trás-os-Montes and Alto Douro campus were peeled and resulting shells (CNS) dried, grounded and sieved at 4 mm mesh before being stored at room temperature.

For CNS pretreatment the following WRF strains were used: UTAD V20 (*Ganoderma resinaceum* Boud.), UTAD 3 (*Irpex lacteus* (Fr.) Fr.), UTAD 156/UF206 (*Phlebia rufa* (Pers.) M. P. Christ.), UTAD 100 (*Bjerkandera adusta* (Willd.) P. Karst.), UTAD 103 (*Trametes versicolor* (L.) Lloyd) and UTAD Tra (*Trametes* sp.). These strains were maintained at 4 °C in potato dextrose agar (PDA) and submitted to periodical subcultures in the Biochemistry Laboratory at UTAD.

Inoculations of CNS with each WRF strain for pretreatment under solid-state fermentation conditions were carried out as previously described by Pinto et al. [17] with minor modifications. Briefly, minimal liquid medium [17] was used to obtain moistened CNS with a 11% (*w*/*v*) solid/liquid ratio. The inoculation was done with four 1 cm^2^ PDA plugs containing fully developed mycelium of each WRF strain. After 21 days of pretreatment, contents of the culture flasks were suspended in 150 mL of deionized water and incubated on a rotary shaker (100 rpm) for 3 h. Extracts were filtered (Whatman GF/A), centrifuged and aliquots were used to determine enzyme activities. Samples of controls and pretreated CNS were washed in order to remove mycelium and soluble molecules, dried at 60 °C until constant weight, and used for further enzymatic saccharification.

### 2.2. Enzyme Assays and CNS Fiber Determination

Absorbance readings were measured at 25 °C using a Helios gamma UV–vis spectrophotometer (Thermo Fischer Scientific, Ashville, NC, USA). Determination of carboxymethylcellulase (CMCase), avicelase and xylanase activities were carried out according to the IUPAC recommendations [20] using 0.5 mL of culture extracts. Briefly, substrates carboxymethylcellulose, avicel and xylan (Sigma, St. Louis, MO, USA) at 1% (*w*/*v*) were prepared in 50 mM citrate buffer, pH 4.8. Hydrolysis was carried out at 50 °C for 30 min for CMCase and xylanase and 180 min for avicelase. Reducing sugars released were determined by dinitrosalicylic acid (DNS) method [21], using glucose as a standard. For ligninolytic enzymes, the activities were monitored as previously described [5,18] using 0.1–0.4 mL of culture extracts and buffered substrates in 1.5 mL reaction volume as shown below. Laccase was measured following the oxidation of 2.0 mM 2,2′-azino-bis (3-ethylbenzthiazoline-6-sulphonic acid) (ABTS) in 100 mM phosphate–citrate buffer pH 4.0 at 420 nm [18]. Manganese peroxidase activity was determined by the formation of Mn^3+^-tartrate from 0.10 mM MnSO_4_, using 100 mM tartrate buffer pH 5 and 0.10 mM H_2_O_2_ [18]. Versatile peroxidase was accessed using 2.0 mM ABTS as substrate in 100 mM tartrate buffer pH 5 and 0.10 mM H_2_O_2_ at 420 nm. Assay values were corrected by subtracting laccase activity (assays without H_2_O_2_) [5]. Lignin peroxidase activity was determined in 100 mM tartrate buffer pH 3.0, monitoring at 310 nm the oxidation of veratryl alcohol in the presence of 0.10 mM H_2_O_2_ [17].

Lignocellulosic composition of CNS was assessed as fiber fractions (dry matter; %DM), viz. ash-free neutral detergent fiber (NDF), ash-free acid detergent fiber (ADF) and acid detergent lignin (ADL) according to van Soest method described by Fernandes et al. [5]. The concentration of cellulose was calculated as the difference between ADF and ADL, and hemicellulose as the difference between NDF and ADF.

### 2.3. Enzymatic Saccharification of Chestnut Shells

Non-fermented CNS (controls) and WRF fermented CNS were hydrolyzed by a commercial enzymatic mix, Onozuka R-10 (Merck, Rahway, NJ, USA) containing CMCase (1 U mg^−1^), xylanase (10 U mg^−1^), β-glucosidase (0.04 U mg^−1^), α-amylase (0.5 U mg^−1^), endo-1,3-β-D-glucanase (0.2 U mg^−1^), pectinase 0.1 U mg^−1^. Saccharification was carried out with two enzymatic mix concentrations, 2.5 U mL^−1^ and 10 U mL^−1^ CMCase activity. CNS samples (0.12 g) of each WRF pretreatment and controls were placed in flasks containing 20 mL of 50 mM citrate buffer, pH 4.8 with 0.01% sodium azide. After swelling overnight, 2 mL of each enzymatic mix concentration were added. Four hydrolysis times: 12, 24, 48 and 72 h at 45 °C with shaking at 100 rpm were used for saccharification analysis. Production of reducing sugars was determined by DNS method [21] using glucose as standard.

### 2.4. Data Processing and Statistical Analysis

Experimental data, with at least three replicates, except in CNS fiber analysis (mean of two replicates), were analyzed by a completely randomized design experiment in one-way ANOVA, using IBM SPSS Statistics 19 (IBM, New York, NY, USA). Multiple linear regression was done in order to analyze the effect of independent or explanatory variables (lignocellulolytic activities in fungal fermentations, CNS hydrolysis time, and concentration of enzymatic hydrolysis mix) on the dependent variable (reducing sugars (rs) production). With this approach, we can analyze which explanatory variables are more likely to be important for modeling the saccharification process. The multiple linear regression general model was: *Y* = *β*_0_ + *β*_1_*ϰ*_1_ + *β*_2_*ϰ*_2_ + …*β_p_*_−1_*ϰ_p_*_−1_ + *ε* where *Y* is the dependent variable (reducing sugars), *β*_0_, *β*_1_, *β*_2_ are the regression parameters; *ϰ*_1_, *ϰ*_2_, *ϰ_p_*_−1_ are the explanatory variables and ε is the random error. Furthermore, the possible interaction between the factor levels was tested, as a model of factorial experiment, with two factors to consider: enzyme concentration/activity (*ϰ*_1_) and time of hydrolysis (*ϰ*_2_), which can be tested using the model: *Y_ijk_* = *µ* + *β*_1_*ϰ*_1*i*_ + *β*_2_*ϰ*_2*j*_ + *β*_3_(*ϰ*_1_*ϰ*_2_)*_ij_* + *ε_ijk_*, where *Y_ijk_* is the observation *k* (reducing sugars value) in level *i* of factor *ϰ*_1_ (enzyme activity or concentration) and level *j* of factor *ϰ*_2_ (time); *µ* is the overall mean; the term *(ϰ*_1_*ϰ*_2_)*_ij_* represent the effect of the interaction in level *i* of factor *ϰ*_1_ with the level *j* of factor *ϰ*_2_, and *ε_ijk_* is the random error. An analysis of covariance was also verified using the model *Y_ij_* = *β*_0_ + *α_i_* + *β*_1_*ϰ_ij_* + *ε_ij_*, where the variability of the dependent variable (reducing sugars) may be explained by defined independent categorical (*α_i_*) and continuous (covariate) *ϰ_ij_* variables.

## 3. Results and Discussion

### 3.1. Fungal Pretreatment of Chestnut Shells: Oxidative and Hydrolytic Activities

Pretreatment of lignocellulosic biomass through fungal solid-state fermentation aims to increase access to structural polysaccharides for subsequent hydrolysis [14,15,16,17,18,19]. In this work, the CNS content of structural polysaccharides and lignin before and after fungal fermentation (Table 1) are in line with those previously reported [8]. WRF mediated degradation of recalcitrant lignin of plant cell walls, involves oxidation reactions catalyzed by laccase, LiP, *MnP* and *VP* enzymes. This group of enzymes, highly versatile in nature, are considered key factors in biomass pretreatment [5,12,13,14,15,16,17,18,19].

**Figure 1:**
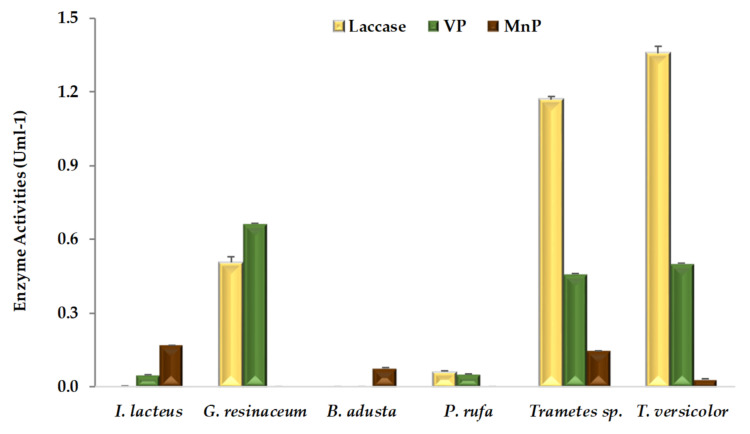
Ligninolytic activities (means ± SD) detected in extracts of chestnut shells after pretreatment by white rot fungal strains.

Extracellular ligninolytic activities detected in WRF-fermented CNS are shown in Figure 1. According to our results, mean laccase activity obtained with all six fungal strains (0.5 U mL^−1^) is much higher than peroxidases, *MnP* and *VP* activities of 0.1 and 0.3 U mL^−1^, respectively, a behavior also observed in solid-state fermentation of CNS in the presence of other fungal species, such as *Coriolopsis rigida* [22]. On the contrary, higher activity for *MnP* than laccase was previously observed during solid-state fermentation of grape stalks [5] and wheat straw [17,18,23]. CNS is a highly lignified substrate even after fungal fermentation (Table 1) and contains a wide range of phenolic acids [2], thus laccase might play a key role, concerning the oxidation of phenolic compounds. In this work, laccase activity of both *Trametes* strains was about twice as high as that of *G. resinaceum*. However, this enzymatic activity was not detected in CNS fermentations with *B. adusta* and *I. lacteus*. Moreover, there is no report of laccase enzyme production from these strains even on the different agro-industrial substrates such as grape stalks [5], wheat straw [17,23], and corn stover [24]. *P. rufa* presented low laccase activity, in line with recent findings in similar conditions, but in the presence of a different substrate [5]. Nevertheless, strong laccase activity, proportional to phenolics concentration, was previously observed in submerged cultures of *P. rufa* [25]. On the other hand, the *Phlebia* genus has been reported as a selective producer of ligninolytic enzymes, promoting efficient bioconversion of lignocellulosic substrates [26].

According to Figure 1, at least one type of peroxidase activity, *MnP* or *VP*, was detected in extracts from each fungal-fermented CNS. While five of the six fungal strains except *B. adusta* produced *VP* enzyme and four fungal strains except for *G. resinaceum* and *P. rufa* were observed to produce *MnP*. None of the fungal strains exhibited LiP activity, although there were previous reports of this enzyme production on grape stalks [5], and wheat straw [17,23], by *B. adusta*, *I. lacteus* [17,18], and *Trametes* sp. [17], respectively. The absence of LiP activity, also observed previously by Dong et al. [27] during fungal CNS pretreatment, was related to the levels of C/N ratio [28] and other external factors such as inactivation by phenolic compounds [29]. Within peroxidases, *VP* firstly reported in fungal genera *Pleurotus* and *Bjerkandera* [30,31], plays an important role in the process of lignin degradation. More recently, *VP* was also isolated from fungal strains of genera *Trametes* [32], *Phlebia* [33], *Ganoderma* [34] and *Irpex* [35]. Taking into account that an efficient lignocellulosic pretreatment enhances enzyme accessibility and subsequent saccharification of structural polysaccharides [10,11,12,36], fungi should excrete an appropriated enzymatic balance between oxidoreductases and hydrolases.

**Figure 2:**
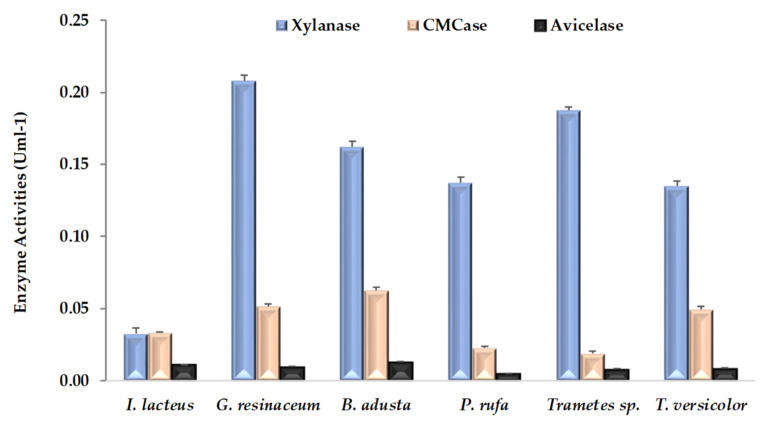
Hydrolytic activities (means ± SD) detected in extracts of chestnut shells after pretreatment by white rot fungal strains.

Three hydrolytic activities accounting for xylanase, CMCase and avicelase were evaluated at the end of CNS pretreatment. As can be seen in Figure 2, all fungal strains produced all of these enzymatic activities, although avicelase values are clearly the lowest. The highest values of xylanase and CMCase activities were detected from CNS fermented by *G. resinceum* and *B. adusta*, respectively. In general, xylanase was a dominant fungal enzyme, being about three to eight times higher than the CMCase activity in all fungal strains except *I. lacteus*. Large differences within enzymatic activities detected in pretreated lignocellulosic substrates have been previously observed in several fungi, including all strains of this work [5,17,18,23]. As previously pointed out [36], this highlights the need to interpret the pattern of enzymatic activities detected as a function of interactions among fungal strains, substrates type and incubation periods.

### 3.2. Saccharification of Pretreated Chestnut Shells

**Figure 3:**
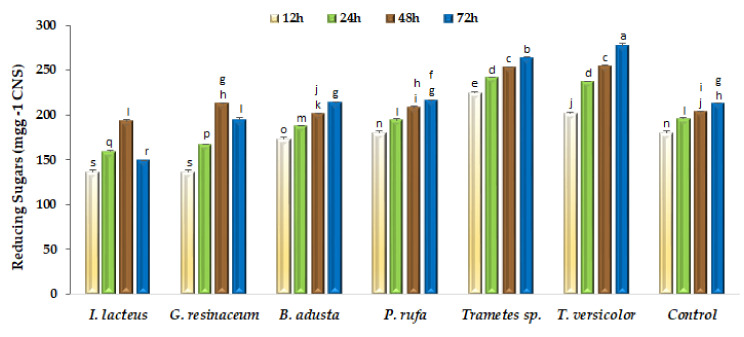
Enzymatic saccharification of non-fermented (Control) and fungal fermented CNS with a commercial preparation containing 10 U mL^−1^ of CMCase activity. Bars with different letters are significantly (*p* < 0.05) different among them.

The saccharification of structural polysaccharides remaining in fermented CNS aims to obtain carbon-neutral soluble sugars for further bioconversion into higher value-added products [12]. Enzymatic hydrolysis of pretreated CNS was evaluated as reducing sugars released at 12, 24, 48 and 72 h (Figure 3). As can be seen, reducing sugars production is clearly influenced by the fungal species used in pretreatment, since some of them did not contribute to increasing saccharification yields, particularly in the first 24 h of hydrolysis. However, after 48 h hydrolysis, both *Trametes* strains and *G. resinaceum* showed significant (*p* < 0.05) saccharification yield increments relative to the non-fermented control. However, only both *Trametes* strains increased the saccharification yield at extended (72 h) hydrolysis time.

Considering biological pretreatments, fungal solid-state fermentations present as major drawbacks during prolonged incubation times, the possibility of sugars consumption as a function of fungal species. According to Figure 3, substrate fermented by fungal strains *I. lacteus* and *G. resinaceum* shows low saccharification yields, which is consistent with higher sugars consumption during the fermentation step and with the observed limited increase of cellulose/lignin ratio. Enzymatic pretreatments can be an alternative but present a major limitation of risk of reaction inhibition by phenolic compounds as well as the high capital cost involved for enzyme production and downstream processing [37]. Thus, solid-state fermentation with selected WRF is an attractive option for the pretreatment of various types of lignocellulosic residues [5,17,23,24,36] and can be important tools in the implementation of circular economy processes for the production of biofuels and other value-added chemicals, according to the concept of biorefinery and the principle of zero waste discharge [38].

### 3.3. Relationship between CNS Saccharification and Explanatory Variables Using Multiple Linear Regression Analysis

Multiple linear regressions were performed to identify which explanatory variables would have the greatest effect on the increase in saccharification in terms of reducing sugars (rs) produced. Thus, we analyzed putative cause-effect relationships between reducing sugars released and dependent variables related to pretreatment and saccharification (lignocellulolytic activities in fungal fermentations, CNS hydrolysis time, and concentration of enzymatic hydrolysis mix). Three statistically significant (*p* < 0.0001) models, A, B, C, (Table 2) were adjusted with high correlations between experimentally observed vs. predicted data.

In model A, the effect of fungal enzymes detected in fermented CNS was evaluated on the saccharification yield after 48 h hydrolysis in the presence of the highest dose of enzymatic hydrolysis mix. Among all fungal enzymes, lignin peroxidase and avicelase were excluded, since their very low values resulted in either *p* > 0.05, or very high estimated regression parameters. When only fungal enzyme activities were used as explanatory variables, (model A, Table 2), we can see that laccase and xylanase activities have positive impacts on increasing CNS saccharification yield. Although laccase and xylanase are not correlated (r = 0.0001), their correlation with saccharification is high for laccase (r = 0.975; *p* < 0.0001) and moderate for xylanase (r = 0.47; *p* < 0.05). A similar effect of xylanase activity on rice straw saccharification was previously reported by Tsujiyama and Ueno [39]. Furthermore, enhanced saccharification of various lignocellulosic substrates due to the action of laccase, alone or in the presence of mediators, has been previously reported by several authors [5,18,40,41]. Conversely, we did not detect positive correlations between both enzymatic activities, peroxidases (*VP* and *MnP*) and CMCase, and the production of reducing sugars (Table 2; models A and B), which is in line with previous observations [39,42]. Furthermore, especially in the presence of high ligninolytic activities, lignin repolymerization and condensation is more likely to occur [43,44] during pretreatment. Thus, the access of cellulolytic enzymes to polysaccharides will be more difficult, which leads to the observed negative correlations between saccharification and these enzymatic activities.

In model B, the effect of low concentration of enzymatic hydrolysis mix was incorporated, and substrate hydrolysis time was maintained at 48 h, as well as the same enzymatic activities. Consistent with the previous model A, laccase and *VP* activities presented (*p* < 0.0001) positive and negative contributions, respectively, to saccharification. Laccase had a main contribution and it seems that *VP* represents a detrimental factor for CNS saccharification. However, the factor “enzymatic hydrolysis mix” is strongly correlated (r = 0.866; *p* < 0.0001) with reducing sugars yield. Model C, in addition to the effect of enzymatic mix concentrations, it also incorporates the effect of substrate hydrolysis time. When factor “CNS hydrolysis time” was taken into account to the reducing sugars production, the effect of two lignocellulolytic activities in fungal fermentations (laccase and xylanase activities) maintained the same behavior. In addition, expected results concerning to the positive effects (*p* < 0.0001) of factors “enzymatic hydrolysis mix” and “CNS hydrolysis time”, are in agreement with a previous work [45].

## 4. Conclusions

Agro-industrial peeling of fruits from *Castanea sativa* produces large amounts of CNS, a recalcitrant and low-value waste. After solid-state fermentation by selected white-rot fungi, this source of polysaccharides can be hydrolyzed into soluble sugars for further fermentation into value-added compounds. In this work, the highest values of reducing sugars production at 48 h hydrolysis occurred in samples fermented by *Trametes* strains (around 253 mg g^−1^ pretreated CNS). Solid-state fermentation performed with both *Trametes* strains allowed to generate significant increments (25% *w*/*w*) in the enzymatic hydrolysis of the pretreated substrate. According to multiple linear regression analysis, our study achieved a high consistency to the positive effects of some variables, namely laccase and xylanase activities during pretreatment, which supports the assumption that they play a key role to increase substrate hydrolysis and reducing sugars yield.

## Figures and Tables

**Table 1:** Chemical composition (% DM) of non-fermented (control) and fungal-fermented CNS.

	NDF	ADF	ADL	Cellulose (a)	Hemic.	Lignin (b)	a/b Ratio
Control	82.7	74.2	38.8	35.5	8.4	38.8	0.9
*I. lacteus*	83.7	78.7	34.0	44.7	5.0	33.7	1.3
*G. resinaceum*	89.8	82.7	46.4	36.3	7.1	46.4	0.8
*B. adusta*	86.0	80.3	39.0	41.3	5.8	39.0	1.1
*P. rufa*	90.5	84.0	44.6	39.4	6.5	44.6	0.9
*T. versicolor*	86.6	82.3	38.9	43.4	4.3	38.9	1.1
*Trametes* sp.	90.5	83.2	41.0	42.3	7.2	41.0	1.0

NDF, neutral detergent fiber; ADF, acid detergent fiber; ADL, acid detergent lignin; Hemic., hemicellulose.

**Table 2:** Estimated regression models for the effect of some dependent variables on the saccharification yield.

Model ^1^	Regression Model	R^2^	Significance
A	rs=203+50×laccase−293×VP−215×MnP+64×xylanase−168CMCase+ε	0.995	*p* < 0.0001
B	rs=40+37×laccase−560×VP+15×enzymatic mix+ε	0.791	*p* < 0.0001
C ^2^	$rs=28+0.72\times \mathrm{hydrolysis}\;\mathrm{time}+15\times \mathrm{enzymatic}\;\mathrm{mix}+23\times \mathrm{laccase}+86\times \mathrm{xylanase}+19\times VP_{cov}-12\times MnP_{cov}+\varepsilon$	0.876	*p* < 0.0001

^1^ A: 48 h of hydrolysis with 10 U mL^−1^ CMCase of enzymatic hydrolysis mix; B: idem with 2.5 U mL^−1^ CMCase of enzymatic hydrolysis mix; C: all hydrolysis times and both concentrations of enzymatic hydrolysis mix. ^2^ $VP_{cov}$ and $MnP_{cov}$ are categorical variables (presence/absence).

## Data Availability

All relevant data are reported in the paper and additional data are available upon request to the authors.
